# A Medical Education for Working Class Children

**Published:** 1919-11-08

**Authors:** 


### THE LATEST SCHOLARSHIPS.

### A Medical Education for Working Class Children.

Under the will of the late Mr. Frank Fletcher, of Sutton Coldfield, provision is made for scholarships for boys or girls who desire to embrace the profession of medicine.

Each scholarship is of the value of £100, and is tenable for five years, and the interesting proviso is that the scholars are to be boys or girls whose parents belong to the working classes, and who are not in receipt of more than 50s. a week. There is no examination laid down, but the scholars are to be elected from pupils attending public elementary schools, or from grammar schools in the case of boys and municipal high schools in the case of girls.

The testator writes :—

"The above provision are framed with a view of affording to children of *bona-fide* wage-earners opportunities of a career in the medical profession, as in my opinion there are potentialities of the born doctor being found amongst such children, in which case it would be in the interests of medical and surgical science, also to the benefit of humanity."

### The First of Their Kind.

Reading it over, it is hard to believe that this is the first time that such a scholarship has been founded, and one cannot but agree with the terms of the will, that should a "born doctor" be found, it would be a great benefit to science and to humanity, but we foresee many difficulties which will need to be overcome before the excellent intentions of the founder can be carried to their realisation.

That there are many children of this class who would make the very best use of such a chance cannot be doubted, in scientific subjects it is far from uncommon to find the "lab boy" who has developed into the head of the laboratory. But would these children be able to develop that catholic interest in persons and things which is so invaluable to the doctor, be he physician or surgeon, as opposed to the purely laboratory worker. The most acute diagnostician, the most brilliant surgeon, is useless unless he possesses the power of attracting and of winning the confidence of his patients.

Arising out of this is the question of the scholar mixing with his or her fellow-students, and with the world in general. The scholarship is worth £100, and with fees, books, and instruments, there will be a very small residue with which the scholar can live and see life in the largest sense of the word. Nor can his family help him, for little can be saved out of 50s. a week, and it must not be forgotten that this boy would have been earning his living, and contributing to the support of the home but for his luck in gaining this scholarship. He may, of course, gain an entrance scholarship at his medical school, and after a short time he may succeed in making some money by coaching, or acting as a student demonstrator, but finance cannot fail to loom very large in his view of life, and this is not a good groundwork.

### The Time Question.

Five years, too, is all too short a date. Who among us in these days qualifies in five years from the time he enters as a student? The writer must confess that he did not, nor did many of his contemporaries.

Nevertheless, we consider this a step in the right direction, and only hope that we may have the pleasure of welcoming a Frank Fletcher scholar at our medical schools. Also we hope that others may take this hint and found more scholarships on the same plan; but if they do, let the scholar receive a living wage in addition to his fees, lest the struggles in which his student years were passed should embitter his life, and in order to prevent also the bounty of the founder of the scholarship being eclipsed by that of the impoverished family of the scholar.

